# Genetic liability to age at first sex and birth in relation to cardiovascular diseases: a Mendelian randomization study

**DOI:** 10.1186/s12920-023-01496-w

**Published:** 2023-04-06

**Authors:** Miao Chen, Zhen Wang, Hongfei Xu, Xiaofang Chen, Peng Teng, Liang Ma

**Affiliations:** Department of Cardiovascular Surgery, The First Affiliated Hospital of Zhejiang University School of Medicine, Number 79 Qingchun Road, Hangzhou, China

**Keywords:** Age at first sex, Age at first birth, Cardiovascular diseases, Mendelian randomization

## Abstract

**Background:**

Growing evidence suggests that various reproductive factors, including early menarche, early menopause, and age at first birth, may increase the risk of developing cardiovascular disease (CVD) later in life. However, the associations between reproductive factors and CVDs are inconsistent and controversial. Therefore, we conducted a two-sample Mendelian randomization (MR) analysis to explore the potential links between age at first sex (AFS) and age at first birth (AFB) and several CVDs.

**Methods:**

We obtained summary statistics for exposure from the largest genome-wide association studies of AFS and AFB. To serve as instrumental variables, we selected 259 SNPs associated with AFS and 81 SNPs associated with AFB at the genome-wide significance level. We employed a random-effects inverse-variance weighted method to pool estimates, and conducted multivariable MR analysis to determine the direct association between AFS and AFB with CVDs, while accounting for the effects of confounders.

**Results:**

The genetic liability to later AFS was associated with decreased risks of heart failure (odd ratio [OR] 0.700; 95% confidence interval [CI] 0.639–0.767; *p* = 2.23 × 10^−14^), coronary artery disease (OR 0.728; 95% CI 0.657–0.808; *p* = 1.82 × 10^−9^), myocardial infarction (OR 0.731; 95% CI 0.657–0.813; *p* = 8.33 × 10^−9^), stroke (OR 0.747; 95% CI 0.684–0.816; *p* = 6.89 × 10^−11^), and atrial fibrillation (OR 0.871; 95% CI 0.806–0.941; *p* = 4.48 × 10^−4^). The genetic liability to later AFB was also associated with decreased risks of CVDs, including myocardial infarction (OR 0.895; 95% CI 0.852–0.940; *p* = 8.66 × 10^−6^), coronary heart disease (OR 0.901; 95% CI 0.860–0.943; *p* = 9.02 × 10^−6^), heart failure (OR 0.925; 95% CI 0.891–0.961; *p* = 5.32 × 10^−5^), and atrial fibrillation (OR 0.944; 95% CI 0.911–0.978; *p* = 0.001). However, no association was found between AFB and stroke. The associations remained independent from the effects of AFS and AFB on potential confounders, including smoking, alcohol intake, body mass index, and depression. Mediation analysis suggested that education attainment partly mediates the link from AFS and AFB to CVD outcomes.

**Conclusion:**

Our results observed a causal relationship between later AFS, AFB and lower CVDs risk; it emphasizes the importance of providing sex education since early sex and birth may have undesirable effects. Cardiovascular risk stratification that considers reproductive factors may help address CVD risk.

**Supplementary Information:**

The online version contains supplementary material available at 10.1186/s12920-023-01496-w.

## Introduction

Human reproductive behaviours, including age at first sexual intercourse (AFS) and age at first birth (AFB), have several important implications for reproductive health [1, 2]. The age of the first sexual intercourse among adolescents has gotten earlier [3]. The survey showed that the median age for first heterosexual intercourse was 17 years in UK teenagers, and the proportion reporting first sexual intercourse before age 16 increased in both sexes [2]. Earlier AFS was related to an increased subsequent risk-take behaviour and a higher likelihood of developing substance use disorders [4, 5]. A phenome-wide study indicated that lower AFS was associated with a higher risk of coronary artery disease (CAD) and was causally associated with several CAD-risk factors, such as low-density lipoprotein, higher fasting insulin, and fasting glucose [6].

In contrast to earlier AFS, women delay pregnancy more frequently in their reproductive years, reaching an average of 30 years in many countries and even later for men [7]. A growing body of evidence supported an association between earlier AFB, cardiovascular diseases and risk factors. A British birth cohort study, including 7600 men and women, indicated that older parents had a lower cardiovascular risk as measured by multiple physiologic characteristics and biochemical markers [8]. Previous studies suggested that younger maternal AFB was associated with increased BMI and a higher risk of obesity and diabetes [9–11]. Furthermore, recent research demonstrated that younger AFB were independently associated with higher systolic and diastolic blood pressure levels [12]. One retrospective study, including over 500,000 participants from UK Biobank, has identified that earlier AFB was independently associated with a higher risk of cardiovascular disease [10].

Despite considerable effort, observational studies were sensitive to confounding bias and reverse causation. For these problems, Mendelian randomization (MR) studies have been advocated to overcome the limitations of conventional studies. MR is a genetic epidemiological method that uses single nucleotide polymorphisms (SNPs) as instrumental variables (IVs) to assess the potential causality of exposure upon outcomes. MR is based on Mendel’s Law of Independent Assortment, wherein alleles are assigned at meiosis, conceptually mimicking a “natural” randomized trial. Recently, several studies explored the potential causal effects of age at menarche and age at menopause on health-related traits by using the MR strategy [13–15]. Multivariable Mendelian randomization is a recently developed methodology that enables simultaneous assessment of correlated exposures by incorporating genetic variations from each risk factor into the same model. Multivariable MR analysis can reduce the influence of confounders when genetic variants are associated with several risk factors. Given the potential confounding and limited causal inference from observational data, we used MR and multivariable MR methods to evaluate the relationships between reproductive behaviours (AFS and AFB) and several cardiovascular diseases.

## Methods and materials

### Study design

We performed a two-sample, summary-based MR analysis to assess the causal relationship of AFS and AFB with cardiovascular diseases using publicly available data. Genetic instrument selection was based on a hitherto largest genome-wide association study (GWAS) for AFS and AFB [16]. Data for the associations of the exposure-associated SNPs with five CVDs were available from corresponding GWASs. Detailed information on the data sources used is available in Additional file 1: Table S1. All original studies included have obtained ethical review approval and informed consent from the participants.

### Data sources and instruments selection

#### Instrumental variables for age at first sexual intercourse and age at first birth

The summary statistics for AFS and AFB were from the largest GWAS meta-analysis [16]. For AFS, the GWAS included 397,338 individuals (n = 182,791 males, n = 214,547 females) from the UK Biobank. For AFB, the GWAS included 542,901 individuals (n = 124,088 males, n = 418,758 females) from 36 studies. AFS is assessed by using questions and treated as a continuous measure. Ages less than 12 are normally excluded. AFB is also treated as a continuous measure, either directly or created from several survey questions. The GWAS identified 282 lead SNPs at genome-wide significance (*p*-value < 5 × 10^−8^) for AFS and 89 lead SNPs at genome-wide significance (*p*-value < 5 × 10^−8^) for AFB. These SNPs explained up to 5.8% of the variance for AFS and 4.8% for AFB [16]. We identified 1000 Genomes proxies for some SNPs and used these in our analysis. We removed SNPs showing a primary association with the outcome rather than the exposure (identified by *p*-value < 5 × 10^−8^ in the outcomes). Any palindromic SNPs that have minor allele frequency above 0.42 were also excluded in our analysis. Finally, 259 SNPs associated with AFS and 81 SNPs associated with AFB were selected as instrumental variables (Additional file 1: Tables S2 and S3). If no matching SNP was available in an outcome GWAS, proxies (linkage disequilibrium r^2^ > 0.60) were found via a search through LD Link (https://analysistools.cancer.gov/LDlink). The detailed information for each proxy is presented in Additional file 1: Table S4.

#### GWAS summary statistics for CVDs outcomes

The GWAS summary statistics for CAD and MI were obtained from the Coronary Artery Disease Genome-Wide Replication and Meta-analysis plus the Coronary Artery Disease Genetics (CardiogramplusC4D) consortium that contained 60,801 cases (43,676 cases with MI) and 123,504 controls [17]. The majority (77%) of the participants were of European ancestry. The GWAS summary statistics of AF were obtained from a GWAS meta-analysis, including 65,446 cases and 522,744 controls from more than 50 studies [18]. We used the datasets of AF only included participants of European descent (55,114 cases and 482,295 controls). The GWAS summary statistics of HF were extracted from a GWAS meta-analysis containing 977,323 (47,309 cases and 930,014 controls) European participants [19]. The stroke data were obtained from a multiancestry GWAS, including 67,162 cases and 454,450 controls [20]. The majority of participants were European, including 40,585 cases and 406,111 controls. Any ischemic stroke (AIS) contained 34,217 cases of European ancestry. The subtypes of ischemic stroke included 4373 large-artery atherosclerotic strokes (LAS) individuals, 5386 small-vessel strokes (SVS) individuals, and 7193 cardioembolic strokes (CES) individuals. In addition to the data for coronary artery disease and myocardial infarction being obtained from mixed populations, we used datasets of other cardiovascular diseases (atrial fibrillation, heart failure, and stroke) only included participants of European descent to minimize confounding by ancestry.

#### Testing instrument strength and statistical power

We calculated the F-statistic for each SNP by using the formula: F-statistic = R^2^ × (N−2)/(1−R^2^) to estimate the strength of genetic instruments [21]. The following formula was used to calculate the R^2^ for each significant SNP: (2 × EAF × (1−EAF) × beta^2^)/(2 × EAF × (1−EAF) × beta^2^ + 2 × EAF × (1−EAF) × N × se^2^), where EAF is the effect allele frequency, N is the sample size, and beta is the estimated effect on AFS or AFB [21]. An F statistic ≥ 10 indicates that the risk of weak instrument bias in MR analyses is relatively low [22]. Statistical power for each outcome was calculated by using the online tool (https://shiny.cnsgenomics.com/mRnd/). Briefly, power is calculated based on the sample size of GWAS, the proportion of cases, and the variance explained by genetic instruments for the exposure.

### Statistical analysis

We used random-effects inverse-variance weighted (IVW) method as the primary statistical analysis. In brief, the data were extracted and harmonized with the direction of estimates. Then we used the formula: Ratio = beta coefficient for the SNP-outcomes association/beta coefficient for the SNP-exposures association to calculate the Ratio estimates for each SNP. These estimates are then combined as standard analysis using the random-effects IVW meta-analysis. Weighted median and MR-Egger regression methods were used as a sensitivity analysis. The weighted median can provide consistent estimates of casual effect if 50% or more of the genetic variants are valid instrumental variables [23]. MR-Egger regression can detect and correct horizontal pleiotropy. The Cochran Q statistic and the I^2^ statistic were performed to assess heterogeneity among estimates across individual SNPs [21]. We considered there was heterogeneity if the *p*-value < 0.05, and we performed I^2^ statistics to quantify heterogeneity (I^2^ ≤ 25%: low heterogeneity; 25% < I^2^ ≤ 50%: moderate heterogeneity; I^2^ ≥ 50%: high heterogeneity).

Multivariable MR analyses were performed to determine whether the effects of AFS and AFB on CVDs may be attenuated by the effects of AFS and AFB on smoking, alcohol, body mass index, depression, and education attainment, which are genetically correlated with AFS and AFB [16]. Smoking, alcohol intake, and body mass index are traditional cardiovascular risk factors. AFS and AFB have strong genetic correlation with major depressive disorder (r_g_ = − 0.37 and − 0.420 respectively) and year of education (r_g_ = 0.53 and 0.74 respectively). Evidence from previous observational clinical trials indicated that depression is a risk factor for CVD incidence and severity [24, 25]. Also, high education may have a potential relationship with a lower risk of CVDs [26–28]. Thus, we conducted multivariable MR adjusting for genetic liability to depression and education attainment.

For the significant MR association, the mediation effects on the pathway from AFS and AFB to CVDs were then estimated using mediation analysis as previously described [29]. First, we used genetic instruments for AFS or AFB to estimate the casual effect of exposures on mediators. Second, we used genetic instruments for mediators to assess the causal effect of identified mediators on CVD outcomes after adjustment for exposures. The mediation effects of exposures on outcomes via potential mediators were assessed by the “product of coefficients” method [29, 30]. Standard errors for the mediation effects were derived by using the delta method [29].

A two-sided *p*-value < 0.05 was considered statistically significant. We adjusted the *p*-value by Bonferroni correction for the number of outcomes. For the primary analysis (association of AFS or AFB with five cardiovascular diseases), the association with a two-sided *p*-value < 0.01 (where α = 0.05/5 outcomes) was deemed statistically significant and a two-sided *p*-value < 0.05 was deemed suggestive. For the secondary analysis (association of AFS with stroke subtypes), we set two-sided p-values of < 0.013 (= 0.05/4 outcomes) as the significance threshold. MR analyses were conducted using the TwoSampleMR (version 0.5.5) and MVMR (version 0.3) in R. All data analyses were conducted with R version 3.6.1.

## Results

### Association of genetic liability to age at first sex with CVDs

**Fig. 1:**
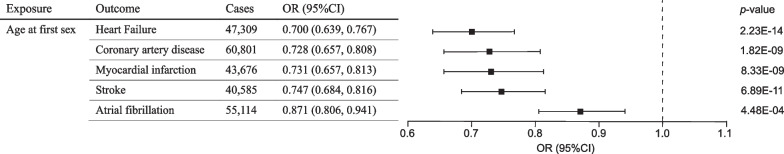
Mendelian randomization association of genetic liability to age at first sex with cardiovascular diseases. OR odds ratio; CI confidence interval. Estimates are from the random-effects inverse variance weighted method. Significant at the Bonferroni-corrected threshold of *p*-value < 0.01

The mean F-statistics for 259 SNPs of AFS was 42.9. For the CVD outcomes, there was > 90% power to detect significant differences at an OR of 0.90 or lower. The genetic liability to AFS was significantly negatively associated with five cardiovascular diseases (Fig. 1). The odds were 0.700 (95% CI 0.639–0.767; *p* = 2.23 × 10^−14^) for heart failure, 0.728 (95% CI 0.657–0.808; *p* = 1.82 × 10^−9^) for coronary artery disease, 0.731 (95% CI 0.657–0.813; *p* = 8.33 × 10^−9^) for myocardial infarction, 0.747 (95% CI 0.684–0.816; *p* = 6.89 × 10^−11^) for stroke, and 0.871 (95% CI 0.806–0.941; *p* = 4.48 × 10^−4^) for atrial fibrillation. Except for atrial fibrillation, results remained consistent and robust in the sensitivity analysis using the weighted median methods (Additional file 1: Table S5). No directional pleiotropy was detected by the MR-Egger approach (Additional file 1: Table S5). There was an indication of moderate heterogeneity measured by I^2^ and Cochran Q for heart failure, coronary artery disease, stroke, and atrial fibrillation and low heterogeneity for myocardial infarction (Additional file 1: Table S6).

We further examined the effects of AFS on ischemic stroke and ischemic stroke subtypes. The genetic liability to late AFS was associated with a lower risk of AIS (OR 0.747; 95% CI 0.682–0.819; *p* = 3.75 × 10^−10^), LAS (OR 0.669; 95% CI 0.545–0.821; *p* = 1.21 × 10^−4^) and SVS (OR 0.755; 95% CI 0.639–0.893; *p* = 9.87 × 10^−4^) (Additional file 1: Table S7). A suggestive association (*p* = 0.034) was found between AFS and CES (OR 0.830; 95% CI 0.699–0.986).

**Fig. 2:**
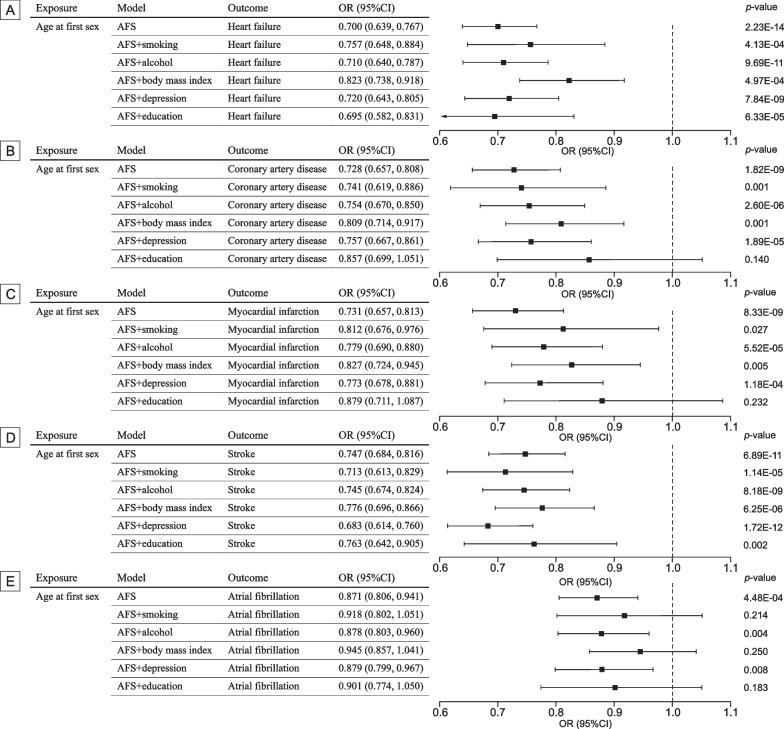
Multivariable Mendelian randomization was performed to estimate the direct effect of the association of age at first sex with cardiovascular disease after accounting for the effects of age at first sex on other confounders

The genetic liability to AFS was significantly associated with smoking, alcohol intake, body mass index, depression and education attainment (Additional file 1: Table S8). In the multivariable MR analysis adjusting for traditional cardiovascular risk factors (smoking, alcohol, and body mass index), the magnitude of associations of genetic liability to AFS with CVDs attenuated, whereas the association of AFS with CVDs remained significant (Fig. 2). The association with atrial fibrillation did not persist after adjusting for smoking and body mass index. Likewise, after adjusting for depression, the overall patterns for the associations of AFS with CVDs remained (Fig. 2). Besides, in the multivariable MR analysis after adjustment for education attainment, most effect sizes of association between AFS and CVDs attenuated mildly and was only the associations with heart failure remained significant (Fig. 2). The mediated proportion of education attainment was 22.6% (95% CI 5.4–39.8%) on heart failure, 42.3% (95% CI 18.3–66.4%) on coronary artery disease, 44.3% (95% CI 18.4–70.3%) on myocardial infarction, and 34.9% (95% CI 14.2–55.7%) on stroke (Additional file 1: Table S9).

In order to examine any potential differences in results between genders, we conducted separate MR analysis for female and male subgroups by utilizing SNPs for AFS. There were 56 SNPs associated with AFS in female groups and 38 SNPs in male groups (Additional file 1: Tables S10 and S11). The results of the MR analysis in the female subgroup were consistent with the primary analysis, except for a suggestive association between AFS and atrial fibrillation (OR = 0.853, 95% CI = 0.753–0.966, *p* = 0.012) (Additional file 1: Table S12). In the male subgroup, however, the genetic liability to AFS was not correlated with coronary artery disease, myocardial infarction, or atrial fibrillation (Additional file 1: Table S13).

### Association of genetic liability to age at first birth with CVDs

**Fig. 3:**
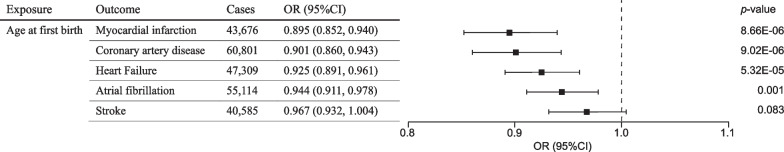
Mendelian randomization association of genetic liability to age at first birth with cardiovascular diseases. OR odds ratio; CI confidence interval. Estimates are from the random-effects inverse variance weighted method. Significant at the Bonferroni-corrected threshold of *p*-value < 0.01

The mean F-statistics for 81 SNPs of AFB was 38.3. There was > 90% power to detect significant differences at an OR of 0.90 or lower. The genetic liability to AFB was significantly negatively associated with myocardial infarction (OR 0.895; 95% CI 0.852–0.940; *p* = 8.66 × 10^−6^), coronary artery disease (OR 0.901; 95% CI 0.860–0.943; *p* = 9.02 × 10^−6^), heart failure (OR 0.925; 95% CI 0.891–0.961; *p* = 5.32 × 10^−5^), and atrial fibrillation (OR 0.944; 95% CI 0.911–0.978; *p* = 0.001) (Fig. 3). There was limited evidence of a negative association between genetically predicted AFS and stroke (OR 0.967, 95% CI 0.932–1.004; *p* = 0.083). Results for the association of AFB with myocardial infarction and coronary artery disease remained consistent and robust in the sensitivity analysis using weighted median methods (Additional file 1: Table S14). There was moderate heterogeneity for CVD outcomes, and no directional pleiotropy was detected (Additional file 1: Tables S14 and S15).

**Fig. 4:**
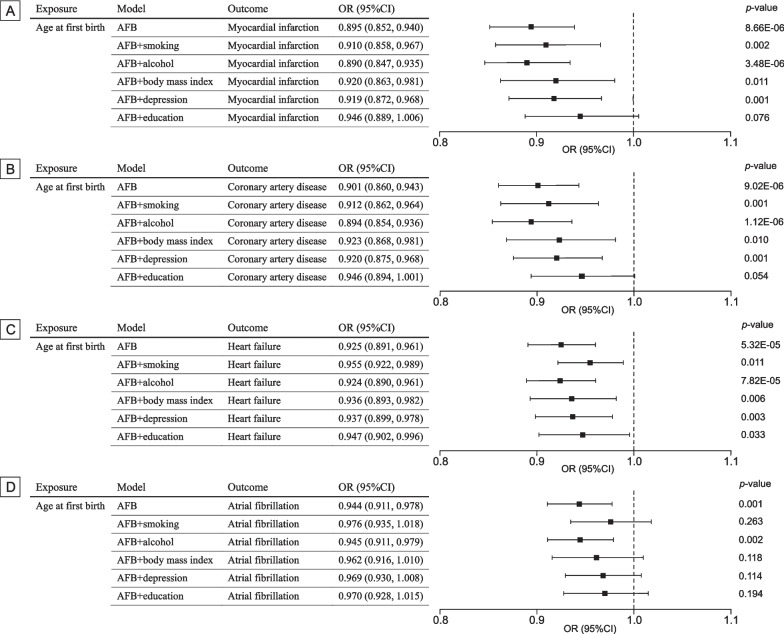
Multivariable Mendelian randomization was performed to estimate the direct effect of the association of age at first birth with cardiovascular disease after accounting for the effects of age at first birth on other confounders

The genetic liability to AFB was also significantly associated with smoking, body mass index, depression and education attainment (Additional file 1: Table S16). In the multivariable MR analysis adjusting for traditional cardiovascular risk factors, overall patterns for the associations of AFB with CVDs remained significant (Fig. 4). However, the association of genetic liability to AFB with atrial fibrillation did not persist after adjusting for smoking and body mass index. The association of genetic liability to AFB with myocardial infarction, coronary artery disease, and heart failure remained significant in the multivariable MR analysis adjusting for depression (Fig. 4). Besides, after adjustment for education, the associations between genetic liability to AFB and CVDs attenuated mildly and failed to reach a significance level (Fig. 4). The mediated proportion of education attainment was 23.9% (95% CI 6.4–41.4%) on myocardial infarction, 25.5% (95% CI 7.8–43.3%) on coronary artery disease, and 25.4% (95% CI 6.4–44.3%) on heart failure (Additional file 1: Table S17).

Similarly, a subgroup analysis of MR for female and male participants was performed using SNPs for AFB. 51 SNPs were found to be associated with AFS in the female group, while no SNP reached genome-wide significance in the male group (Additional file 1: Table S18). The female subgroup MR analysis showed consistency with the primary analysis, with a suggestive association between AFB and myocardial infarction observed (OR = 0.928, 95% CI = 0.876–0.984, *p* = 0.012) (Additional file 1: Table S19).

## Discussion

Using the MR approach, we utilize population-scale human genetics to evaluate a potentially causal association between AFB, AFS, and several cardiovascular diseases (coronary artery disease, myocardial infarction, heart failure, atrial fibrillation, and stroke). By extracting summary statistics from several large GWASs, we could consider more cases by order of magnitude for AFB, AFS, and cardiovascular outcomes compared with previous observational studies. Residual confounding is one limitation of observational studies that may bias the estimates. The genetic instrument variables for AFB and AFS used in this study provided estimates less influenced by reverse causality and confounding factors. Because alleles are assigned at meiosis, MR conceptually mimicked a "natural" randomized trial, and we were able to estimate potentially causal relationships between AFB, AFS and cardiovascular diseases.

The present study based on a GWAS of AFS (n = 125,667) and AFB (n = 343,072) found that genetic liability to late AFS and AFB were associated with a lower risk of a broad range of CVDs. The strongest associations were observed for coronary artery disease, myocardial infraction, heart failure. There was suggestive association for atrial fibrillation. The evidence supported a strong association between AFS and stroke but no association between AFB and stroke. AFS and AFB had a high genetic correlation with body mass index, alcohol intake, and smoking, which were well-known cardiovascular risk factors. Further, AFS and AFB were also genetically associated with depression and education attainment, which may be a confounder in the observed association between AFB, AFS and CVDs. The pattern of most associations remained significant after adjusting for smoking, alcohol, body mass index, and depression. However, in the multivariable MR models adjusting for education, the magnitude of most associations of attenuated mildly and these association did not persist, which suggested that education attainment might partly mediate the link from AFS and AFB to these CVDs.

A recent comprehensive phenome-wide search indicated that genetic variants to higher cognition were associated with lower risks of CVDs, and this trend is in part attributed to higher AFS. They observed that lower AFS was concordantly associated with a higher risk of CVD in both males and females; lower AFS was causally associated with several CVD-risk factors. Our MR analysis further confirmed the potentially causal association between AFS and several CVDs by using the latest available and largest GWAS summary data of AFS. Many reproductive health-related factors, such as pre-eclampsia, stillbirth, preterm birth, early menarche, polycystic ovary syndrome, ever parity, and early menopause, were associated with cardiovascular diseases [31]. However, epidemiological evidence on the associations of AFS with cardiovascular diseases is scarce. Although our MR analysis strongly supports a potentially causal relationship between late AFS and lower risks of several cardiovascular diseases, further clinical evidence is needed to validate this association.

Previous studies observed that teenage motherhood was associated with an increased risk of all-cause mortality [32–34] and cardiovascular mortality [35, 36]. When looking at specific CVD outcomes, the majority of clinical observational studies identified that AFB was inversely associated with coronary heart disease [37, 38], myocardial infarction [39], and stroke [37, 40]; our MR results provided evidence that may be consistent with a potential causal relationship. In a previous analysis that used prospective data from 482,000 women and men in the UK Biobank, an inverse relationship between AFB and the risk of CVDs (adjusted HRs for every year increase in AFB: 0.97, 95% CI 0.96–0.98), coronary heart disease (adjusted HRs: 0.96, 0.95–0.97) and stroke (adjusted HRs: 0.98, 0.97–0.99) was found [37]. Similarly, a statistically significant inverse association of ischemic stroke incidence with later AFB (age ≥ 25 versus age 21–24 years: HR = 0.6, 0.4–0.9) was reported in a cohort study of 45,699 middle-aged women [40]. However, our results did not support a assocaiton between AFB and storke. The clinical evidence on the associations of AFB with atrial fibrillation and heart failure was scarce. Several lines of secondary evidence may lead to support these novel findings. In detail, studies revealed that early AFB (younger than 20 years) was associated with several cardiovascular factors by mid-life, including body mass index, obesity, blood pressure, cholesterol, triglycerides, glycated haemoglobin, C reactive protein, von Willebrand factor, and fibrinogen [8–10, 12].

The original GWAS of AFS and AFB also examined their influence on CAD through multivariable MR that adjusted for years of education [16]. The results indicate significant associations between AFS and AFB with CAD, which were significantly attenuated by adjusting for years of education. A recent study by Wang et al. conducted a MR analysis that focuse on explore the causal relationship between women's reproductive traits and ischemic stroke, taking into consideration the potential influence of body mass index and educational attainment [41]. They found consistent and significant associations between earlier AFB and AFS and an increased risk of ischemic stroke, in both female-only and sex-combined analyses. Compared to the above two studies, our study evaluated the causal relationship between AFS and AFB and a diverse range of CVDs while adjusting for several modifiable risk factors. Although some results are consistent with previous studies, our research further discovered the causal relationship between AFS and AFB with atrial fibrillation, heart failure, and myocardial infarction.

The exact pathological mechanism between later AFS, AFB and decreased risk of CVDs was presently unclear. Several plausible biological pathways supported these associations. On the one hand, women who had conceived pregnancy before the age of 20 years were more likely to develop pregnancy-related complications, including hypertension, eclampsia, lasting insulin resistance and altered cholesterol profiles [42, 43]. Some findings suggested that these pregnancy-related complications might contribute significantly to CVDs developments [44]. On the other hand, AFS and AFB correlate with socioeconomic position and behavioural factors, including physical activity, smoking, alcohol intake and diet. Later parenthood might be related to greater control for health behaviours, such as smoking and alcohol intake, which influence CVDs development [45]. An alternative explanation for the association of AFS and AFB with later health is that early parenthood may lead to reduced educational attainment and financial resources and change career trajectories [43–45], resulting in stress and poorer health. Nevertheless, early life circumstances, such as pubertal health and parental separation, might potentially confound the association of AFS and AFB with later health. Therefore, relatively little is known about why AFB and AFB were associated with later health.

One significant strength of our study over previous studies was the great sample size in outcomes GWAS provided considerable statistical precision and accuracy. Observational studies are unavoidably subjected to confounding bias that may lead to spurious associations. MR design, especially multivariable MR analysis, reduced the impact of confounders. Furthermore, the statistical power for the analyses of AFS and AFB with cardiovascular outcomes was high. Nevertheless, the present study must be interpreted within the context of some limitations. First, we primarily focused on individuals of European ancestry in exposure and outcome datasets, so the population stratification bias was minimized, but it may limit generalization to other populations. Second, MR required a critical assumption that genetic association with the outcomes was through the exposure of interest and not via other pathways. Although findings were consistent in sensitivity analysis and adjusted for several cardiovascular risk factors, we could not entirely exclude the possibility that AFS and AFB influence CVDs through other factors and pathways. Third, the present study used linear MR analyses and showed that AFS and AFB were negatively associated with CVDs. We considered that nonlinear MR analyses need to be performed using stratified datasets (early and later age at first sex and birth) to further confirmed the association of AFS and AFB with CVDs. Finally, we conducted sex-specific MR analyses using available exposure data for AFS and AFB in men and women separately, and utilizing available outcome data from both sexes. However, it is better to analyze CVD outcomes separately for men and women.

In conclusion, the present study using large genetic data supported a potentially causal relationship of genetic liability to later AFS and AFB with lower risks of several CVDs. Our finding suggested that it is important to provide sex education since early sex and birth may have undesirable effects. More frequent cardiovascular screening among individuals who have early sex or birth might help to prevent onset of CVDs.

## Supplementary Information

**Additional file 1**. Supplementary Tables of Genetic liability to age at first sex and birth in relation to cardiovascular diseases: A Mendelian Randomization study.

## Data Availability

Our study used publicly available summary-level data of GWAS. The summary statistics for age at first birth and age at first sex are available on the GWAS catalogue website: https://www.ebi.ac.uk/gwas/downloads/summary-statistics. The data for education attainment is available at the website http://www.thessgac.org/data. The summary statistics of GWAS for atrial fibrillation, heart failure, coronary artery disease, myocardial infarction, stroke, any ischemic stroke, large-artery stroke, small-vessel stroke, cardioembolic stroke, smoking initiation, alcohol, body mass index, and depression can be accessed at IEU OpenGWAS project (https://gwas.mrcieu.ac.uk/); Each IDs were presented in Additional file 1: Table S1. The codes used in this study are available from the corresponding author on reasonable request.
