# Supplementary material for: Genetic liability to age at first sex and birth in relation to cardiovascular diseases: a Mendelian randomization study
Source: BMC Med Genomics. 2023 Apr 6;16:75. doi: 10.1186/s12920-023-01496-w (PMC10080931; doi:10.1186/s12920-023-01496-w)
Supplement: Supplementary file 1 — Additional file 1. Supplementary Tables of Genetic liability to age at first sex and birth in relation to cardiovascular diseases: A Mendelian Randomization study. [file 12920_2023_1496_MOESM1_ESM.pdf]

Genetic liability to age at first sex and birth in relation to cardiovascular diseases: A Mendelian Randomization study

Supplementary material

Table 1. Descriptive information of the studies and datasets included in the analyses.

GWAS	Phenotype	Participants	Ancestry	Use in this MR study	ID in IGD*
Mills MC, 2021	Age at first sex	182,791 males; 214,547 females	European	Exposure	NA.
Mills MC, 2021	Age at first birth	124,088 males; 418,758 females	European	Exposure	NA.
Roselli C, 2018	Atrial fibrillation	55,114 cases; 482,295 controls	European	Outcome	ebi-a-GCST006061
Shah S, 2020	Heart failure	47,309 cases; 930,014 controls	European	Outcome	ebi-a-GCST009541
CARDIoGRAMplusC4D	Coronary artery disease	60,801 cases; 123,504 controls	Multi-ancestry	Outcome	ieu-a-7
CARDIoGRAMplusC4D	Myocardial infarction	43,676 cases; 128,199 controls	Multi-ancestry	Outcome	ieu-a-798
Malik R, 2018	Stroke	40,585 cases; 406,111 controls	European	Outcome	ebi-a-GCST005838
Malik R, 2018	Any ischemic stroke	34,217 cases; 406,111 controls	European	Outcome	ebi-a-GCST005843
Malik R, 2018	Large-artery stroke	4,373 cases; 406,111 controls	European	Outcome	ebi-a-GCST005840
Malik R, 2018	Small-vessel stroke	5,386 cases; 192,662 controls	European	Outcome	ebi-a-GCST005841
Malik R, 2018	Cardioembolic stroke	7,193 cases; 406,111 controls	European	Outcome	ebi-a-GCST005842
GSCAN	Smoking initiation	1,232,091 individuals	European	Confounder	ieu-b-4877
GSCAN	Alcohol	941,280 individuals	European	Confounder	ieu-b-73
GIANT	Body mass index	322,154 individuals	Multi-ancestry	Confounder	ieu-a-2
Howard DM, 2019	Depression (excluding 23andme)	170,756 cases, 329,443 controls	European	Confounder	ieu-b-102
Lee JJ, 2018	Education attainment	1,131,881 individuals	European	Confounder	NA.

*IGD: The IEU GWAS database (https://gwas.mrcieu.ac.uk/)

Table 2. Associations of single nucleotide polymorphisms for age at first sex.

SNP	Chr	Position	EA	NEA	EAF	beta	SE	*p*-value	N*	F-statistic
rs1962545	1	7522336	T	C	0.4799	0.0124	0.0021	4.20E-10	387338	34.9
rs803679	1	44349405	G	A	0.2055	-0.0175	0.0025	6.80E-12	387338	49.0
rs7533341	1	58255002	G	C	0.3878	-0.0119	0.0021	1.30E-08	387338	32.1
rs1392816	1	66481188	C	T	0.6125	-0.017	0.0021	6.20E-16	387338	65.5
rs1119919	1	72887402	C	A	0.9269	-0.0246	0.0041	2.30E-09	387338	36.0
rs7721450	1	75316394	A	T	0.9519	-0.0315	0.0048	3.10E-11	387338	43.1
rs3451743	1	78450517	C	A	0.8728	0.0196	0.0032	6.50E-10	387338	37.5
rs1416550	1	87794596	C	T	0.9612	0.0301	0.0054	2.70E-08	387338	31.1
rs1156981	1	88829969	A	G	0.0985	0.0207	0.0035	1.60E-09	387338	35.0
rs1931262	1	96174912	T	C	0.5092	0.0118	0.0021	1.00E-08	387338	31.6
rs1146566	1	97122384	A	G	0.2273	0.0133	0.0025	6.90E-08	387338	28.3
rs2274568	1	110612925	G	A	0.4198	0.0129	0.0021	4.60E-10	387338	37.7
rs7976448	1	115330070	G	A	0.6672	-0.0127	0.0022	1.10E-08	387338	33.3
rs1120477	1	151054180	G	A	0.2197	-0.0151	0.0025	1.90E-09	387338	36.5
rs4612664	1	153822673	T	C	0.6776	0.0141	0.0022	2.60E-10	387338	41.1
rs1476337	1	156320524	C	T	0.9339	0.0242	0.0042	8.10E-09	387338	33.2
rs1997699	1	198219387	G	C	0.8429	0.0173	0.003	3.80E-09	387338	33.3
rs1124033	1	204968339	C	T	0.7383	-0.014	0.0024	2.20E-09	387338	34.0
rs1015716	1	214484950	C	T	0.5835	0.0119	0.0021	1.20E-08	387338	32.1
rs2275307	1	232574921	T	C	0.7518	-0.0134	0.0024	2.10E-08	387338	31.2
rs6586405	1	234739101	C	A	0.6720	0.0122	0.0022	3.00E-08	387338	30.8
rs1320330	2	622225	T	G	0.1717	0.0178	0.0027	1.10E-10	387338	43.5
rs2091377	2	6145158	C	T	0.6269	-0.0118	0.0021	3.10E-08	387338	31.6
rs2014149	2	14539477	T	C	0.4746	0.012	0.0021	1.20E-08	387338	32.7
rs6744657	2	22554648	A	G	0.6310	0.0144	0.0021	1.30E-11	387338	47.0
rs1246372	2	26948413	G	A	0.4734	-0.0141	0.0021	5.90E-12	387338	45.1
rs4952343	2	32858637	A	G	0.5528	-0.0126	0.0021	2.20E-09	387338	36.0
rs5625488	2	44842184	T	A	0.3803	0.0191	0.0021	1.40E-19	387338	82.7
rs985919	2	50687013	C	A	0.7107	0.0143	0.0023	2.20E-10	387338	38.7
rs1516172	2	51853955	C	G	0.7987	-0.0155	0.0026	1.70E-09	387338	35.5
rs9789483	2	58492345	A	G	0.6346	0.0131	0.0021	2.20E-09	387338	38.9
rs6719762	2	60166832	T	C	0.5257	0.0221	0.0021	7.20E-27	387338	110.7
rs359243	2	60475509	T	C	0.3925	0.0213	0.0021	1.10E-23	387338	102.9
rs1232834	2	60785937	A	G	0.5581	-0.0135	0.0021	2.00E-10	387338	41.3
rs6218026	2	63320524	T	C	0.7809	-0.0196	0.0025	1.40E-15	387338	61.5
rs1168802	2	78018164	A	G	0.9266	-0.0224	0.004	1.40E-08	387338	31.4
rs1368546	2	104057364	T	C	0.4433	-0.0165	0.0021	2.50E-15	387338	61.7
rs4583397	2	140328261	T	C	0.3692	-0.0161	0.0022	3.50E-13	387338	53.6
rs1269259	2	161265910	C	T	0.6276	0.0119	0.0021	2.40E-08	387338	32.1
rs1167898	2	162101261	G	A	0.5373	0.012	0.0021	1.20E-08	387338	32.7

rs1016588	2	164879903	C	A	0.7798	-0.0138	0.0025	7.90E-09	387338	30.5
rs1338797	2	166193965	C	A	0.5275	0.0119	0.0021	9.80E-09	387338	32.1
rs1300932	2	171624415	A	T	0.6696	0.0118	0.0022	4.00E-08	387338	28.8
rs7575189	2	174015168	G	A	0.4107	-0.0164	0.0021	1.90E-15	387338	61.0
rs5630605	2	184416610	G	A	0.7853	-0.0157	0.0025	4.10E-10	387338	39.4
rs1477251	2	185529344	C	T	0.9610	0.0307	0.0054	5.70E-09	387338	32.3
rs1300648	2	212691559	A	C	0.5173	-0.0131	0.0021	6.90E-10	387338	38.9
rs875097	2	220353104	A	G	0.2988	0.0125	0.0023	3.40E-08	387338	29.5
rs6748341	2	225377574	C	G	0.6847	-0.0149	0.0022	1.10E-11	387338	45.9
rs9809849	3	3726156	G	A	0.5747	0.014	0.0021	1.80E-11	387338	44.4
rs2084572	3	17315758	A	G	0.5492	-0.014	0.0021	3.50E-11	387338	44.4
rs6550942	3	19115331	C	T	0.7846	-0.0137	0.0025	3.00E-08	387338	30.0
rs1144563	3	24673158	C	A	0.9803	-0.0441	0.0075	1.00E-08	387338	34.6
rs2362774	3	24920083	A	G	0.3918	0.0124	0.0021	1.80E-08	387338	34.9
rs2278480	3	25635743	T	C	0.7124	-0.0125	0.0023	2.70E-08	387338	29.5
rs6772342	3	35775115	T	A	0.6243	-0.0146	0.0021	1.30E-11	387338	48.3
rs1115990	3	49502779	A	G	0.1144	0.0316	0.0034	6.60E-21	387338	86.4
rs2188151	3	50201924	G	T	0.5745	0.021	0.0021	2.10E-24	387338	100.0
rs2612029	3	53773492	T	C	0.1621	-0.0239	0.0028	1.20E-17	387338	72.9
rs1867234	3	54156598	A	G	0.8483	0.0183	0.0029	5.10E-11	387338	39.8
rs6764919	3	60879538	G	A	0.7367	0.0135	0.0023	2.30E-09	387338	34.5
rs6445264	3	62354425	G	A	0.3307	0.0126	0.0023	4.50E-08	387338	30.0
rs7618715	3	70873278	G	A	0.5808	-0.0126	0.0021	1.90E-09	387338	36.0
rs4334682	3	74949456	T	C	0.1559	0.0185	0.0029	1.20E-10	387338	40.7
rs1271459	3	84387950	A	C	0.7252	0.0217	0.0023	1.10E-20	387338	89.0
rs1125235	3	85650341	T	C	0.3717	-0.024	0.0022	1.20E-28	387338	119.0
rs6797231	3	86671515	G	C	0.6880	0.0163	0.0022	2.10E-13	387338	54.9
rs1271470	3	88249922	A	G	0.1590	0.0242	0.0028	1.40E-17	387338	74.7
rs9819555	3	94006243	G	A	0.4349	-0.0123	0.0021	1.60E-09	387338	34.3
rs1377276	3	107948019	C	G	0.4165	0.0183	0.0021	3.00E-18	387338	75.9
rs1769913	3	115885360	G	A	0.7582	0.0131	0.0025	4.60E-08	387338	27.5
rs5794512	3	117641724	C	T	0.8511	-0.0229	0.0029	6.40E-15	387338	62.4
rs705240	3	118457615	C	T	0.8150	0.015	0.0027	7.00E-09	387338	30.9
rs5639224	3	131968209	A	C	0.6068	0.014	0.0021	8.90E-12	387338	44.4
rs1685945	3	147161075	A	G	0.6146	-0.0138	0.0023	1.90E-09	387338	36.0
rs7785711	4	19299307	C	G	0.6693	0.0148	0.0022	9.70E-12	387338	45.3
rs3481147	4	25408838	G	A	0.7683	-0.0155	0.0024	1.30E-10	387338	41.7
rs702	4	28710551	A	T	0.1622	-0.0181	0.0028	2.80E-10	387338	41.8
rs7263106	4	60247508	C	T	0.9698	-0.035	0.006	4.40E-09	387338	34.0
rs7671317	4	62972597	G	T	0.3185	-0.0122	0.0022	3.50E-08	387338	30.8
rs993700	4	67825894	T	C	0.2225	-0.0157	0.0025	2.90E-10	387338	39.4
rs1051687	4	91590266	T	G	0.7653	0.0148	0.0024	6.60E-10	387338	38.0

rs1172908	4	112503872	G	A	0.8278	-0.0222	0.0027	4.10E-16	387338	67.6
rs809955	4	140874760	G	A	0.6340	-0.0157	0.0021	1.00E-13	387338	55.9
rs435538	5	24921398	C	G	0.7691	0.017	0.0024	2.50E-12	387338	50.2
rs3435706	5	30041736	G	T	0.6337	0.0122	0.0022	3.10E-08	387338	30.8
rs1251743	5	30842054	T	G	0.4577	0.0123	0.0021	3.30E-09	387338	34.3
rs1252339	5	45119647	T	A	0.8275	-0.0219	0.0027	1.10E-15	387338	65.8
rs4865723	5	49440206	T	C	0.2652	-0.0146	0.0026	4.40E-09	387338	31.5
rs6449608	5	50696922	T	A	0.6171	-0.0123	0.0021	6.80E-09	387338	34.3
rs7381195	5	60030791	T	A	0.3886	0.0136	0.0021	1.60E-10	387338	41.9
rs1005540	5	77628709	A	G	0.6614	0.0124	0.0022	3.30E-08	387338	31.8
rs5777228	5	104757151	T	C	0.6013	-0.0126	0.0021	2.00E-09	387338	36.0
rs2406374	5	106936435	C	T	0.6856	-0.0139	0.0022	4.30E-10	387338	39.9
rs1195925	5	112018894	A	G	0.8203	-0.0148	0.0027	4.80E-08	387338	30.0
rs1005836	5	124235427	A	G	0.8221	-0.0179	0.0028	1.90E-10	387338	40.9
rs2910032	5	152540354	C	T	0.4821	-0.0128	0.0021	3.00E-10	387338	37.2
rs1298310	5	154900639	G	T	0.6863	0.0163	0.0022	3.00E-13	387338	54.9
rs292423	5	163053445	G	A	0.5545	0.0122	0.0021	7.30E-09	387338	33.8
rs4868800	5	166985224	G	T	0.4183	-0.0128	0.0021	8.20E-10	387338	37.2
rs1195543	5	167420296	T	G	0.6955	0.0137	0.0023	1.40E-09	387338	35.5
rs245753	5	170486143	T	C	0.3182	0.0133	0.0022	1.60E-09	387338	36.5
rs1220359	6	396321	C	T	0.7803	-0.014	0.0026	3.60E-08	387338	29.0
rs2744042	6	19183178	A	G	0.2271	-0.0149	0.0025	2.20E-09	387338	35.5
rs767943	6	23446691	C	A	0.7375	0.0171	0.0024	5.70E-13	387338	50.8
rs766406	6	26319588	G	T	0.3908	-0.0136	0.0021	2.30E-10	387338	41.9
rs7746553	6	31895973	C	G	0.8485	0.0161	0.0029	4.90E-08	387338	30.8
rs1415477	6	50615935	G	A	0.9179	-0.0324	0.0038	1.40E-17	387338	72.7
rs222440	6	52946320	T	C	0.1802	-0.017	0.0027	4.90E-10	387338	39.6
rs2653349	6	55142337	G	A	0.7663	0.0139	0.0025	3.40E-08	387338	30.9
rs1925686	6	87858691	G	A	0.6107	-0.0123	0.0021	7.30E-09	387338	34.3
rs2397678	6	100345238	A	G	0.3064	0.0154	0.0022	1.70E-11	387338	49.0
rs7739574	6	101380707	C	T	0.2667	-0.0137	0.0024	6.70E-09	387338	32.6
rs7299085	6	105147784	G	A	0.9038	-0.0215	0.0035	8.50E-10	387338	37.7
rs7452074	6	125050993	A	C	0.4739	0.0129	0.0021	4.50E-10	387338	37.7
rs1321687	6	145257393	A	G	0.8838	0.0175	0.0032	4.40E-08	387338	29.9
rs1220471	6	152235339	C	T	0.3687	-0.0275	0.0021	8.30E-38	387338	171.5
rs205983	6	164422743	C	G	0.3646	0.0138	0.0022	4.10E-10	387338	39.3
rs6951209	7	1273821	T	A	0.2246	0.0201	0.0025	2.60E-15	387338	64.6
rs4552798	7	1928001	C	T	0.4622	-0.0164	0.0021	7.40E-15	387338	61.0
rs7785195	7	3424686	G	A	0.3409	-0.0142	0.0022	9.50E-11	387338	41.7
rs198310	7	24191861	A	T	0.7862	-0.0168	0.0025	3.00E-11	387338	45.2
rs3585155	7	31330785	A	G	0.8983	0.0247	0.0034	2.50E-13	387338	52.8
rs1023347	7	32265007	C	T	0.7482	0.0142	0.0024	4.20E-09	387338	35.0

rs1270126	7	32962089	C	T	0.6001	-0.0126	0.0021	5.90E-09	387338	36.0
rs794375	7	75147801	T	C	0.5728	-0.0142	0.0021	1.40E-11	387338	45.7
rs6962772	7	99081730	A	G	0.8459	-0.0228	0.0029	2.10E-15	387338	61.8
rs1330722	7	105084671	G	A	0.1070	-0.0218	0.0034	4.70E-11	387338	41.1
rs2694934	7	113973708	A	G	0.2487	-0.0175	0.0024	9.20E-13	387338	53.2
rs7783012	7	114116881	G	A	0.4072	0.0196	0.0021	1.40E-20	387338	87.1
rs1177016	7	116417848	G	C	0.6685	0.0139	0.0022	3.50E-10	387338	39.9
rs4730883	7	118299289	C	A	0.4908	-0.0113	0.0021	4.50E-08	387338	29.0
rs1176728	7	121947456	A	G	0.7788	0.0191	0.0025	2.80E-14	387338	58.4
rs1177244	7	133453874	G	A	0.8112	0.0203	0.0027	2.10E-14	387338	56.5
rs6966898	7	135221170	C	T	0.6681	0.0132	0.0022	1.60E-09	387338	36.0
rs1133672	7	140144414	C	T	0.7207	-0.0172	0.0023	1.90E-13	387338	55.9
rs1991651	8	10706411	C	G	0.3824	-0.0182	0.0021	1.70E-17	387338	75.1
rs7008955	8	26334103	T	G	0.4727	-0.0116	0.0021	3.40E-08	387338	30.5
rs7828172	8	38340137	A	G	0.4000	-0.0119	0.0021	1.40E-08	387338	32.1
rs7824756	8	51118559	T	C	0.7062	0.0155	0.0023	2.80E-11	387338	45.4
rs4537316	8	53131574	T	C	0.8229	0.0142	0.0027	4.60E-08	387338	27.7
rs1585634	8	54396376	G	C	0.1988	0.0146	0.0026	1.30E-08	387338	31.5
rs1010452	8	73889570	T	C	0.4777	0.0123	0.0021	6.70E-10	387338	34.3
rs6997641	8	86676101	C	T	0.1739	-0.0182	0.0027	1.50E-11	387338	45.4
rs3591843	8	91894514	T	C	0.7430	0.0137	0.0024	1.30E-08	387338	32.6
rs3724201	8	93394601	C	T	0.9786	-0.0405	0.0072	3.10E-08	387338	31.6
rs7267482	8	95489281	T	C	0.7587	-0.0137	0.0024	3.40E-08	387338	32.6
rs1095508	8	97825208	C	T	0.4213	0.0131	0.0021	2.50E-10	387338	38.9
rs9643087	8	115382484	C	T	0.4750	0.0135	0.0021	9.40E-11	387338	41.3
rs1328059	8	116686752	C	G	0.2731	0.0134	0.0023	9.30E-09	387338	33.9
rs8180995	8	143326237	A	G	0.5350	-0.0122	0.0021	8.90E-10	387338	33.8
rs7033296	9	8932704	A	T	0.2002	0.0149	0.0026	1.60E-08	387338	32.8
rs4961705	9	16347927	G	C	0.6528	-0.013	0.0022	4.20E-09	387338	34.9
rs1255451	9	23352293	T	C	0.5834	-0.0126	0.0021	1.20E-09	387338	36.0
rs1074657	9	81512820	A	G	0.6604	-0.0129	0.0022	5.40E-09	387338	34.4
rs3501078	9	86499923	G	A	0.8735	-0.0194	0.0032	1.70E-09	387338	36.8
rs1099281	9	96392182	G	A	0.6288	-0.0121	0.0022	1.70E-08	387338	30.2
rs2090409	9	108967088	C	A	0.6523	0.0153	0.0022	2.60E-12	387338	48.4
rs9886840	9	124602728	A	G	0.4203	-0.0115	0.0021	4.40E-08	387338	30.0
rs4292819	9	134929122	T	C	0.3188	-0.0147	0.0022	2.10E-11	387338	44.6
rs7358158	9	140251458	G	A	0.8762	0.0171	0.0031	1.70E-08	387338	30.4
rs1125590	10	8798182	A	C	0.7444	0.0135	0.0024	1.20E-08	387338	31.6
rs1159917	10	9982177	C	A	0.6160	-0.0126	0.0021	2.80E-09	387338	36.0
rs2093623	10	10922977	G	A	0.5030	-0.0139	0.0021	4.50E-11	387338	43.8
rs2650705	10	63239803	A	G	0.1621	0.0166	0.0028	3.70E-09	387338	35.1
rs6185697	10	97941022	T	C	0.6600	0.0154	0.0022	5.70E-13	387338	49.0

rs1224438	10	104640052	G	A	0.6626	0.0136	0.0022	5.80E-10	387338	38.2
rs3896224	10	106467853	A	G	0.5877	-0.0198	0.0021	2.70E-21	387338	88.9
rs1074923	10	118777998	G	C	0.2363	-0.0179	0.0025	4.90E-13	387338	51.3
rs6186445	10	120001867	A	G	0.8178	-0.0147	0.0027	2.50E-08	387338	29.6
rs7897631	10	127176961	A	G	0.4560	-0.0133	0.0021	1.70E-10	387338	40.1
rs7079070	10	134182921	G	A	0.5463	0.0165	0.0021	1.20E-15	387338	61.7
rs1866710	11	12875312	A	G	0.2919	-0.0148	0.0023	2.30E-10	387338	41.4
rs1083538	11	28671629	G	C	0.6554	-0.0161	0.0022	6.60E-14	387338	53.6
rs3480422	11	43771084	A	G	0.5936	-0.0151	0.0021	1.90E-13	387338	51.7
rs6188275	11	46611805	A	G	0.7382	0.0166	0.0026	2.20E-10	387338	40.8
rs1104036	11	49450176	G	A	0.1486	-0.0192	0.0031	5.30E-10	387338	38.4
rs4439537	11	79887549	T	C	0.4763	-0.0132	0.0021	1.10E-10	387338	39.5
rs7943198	11	112882179	T	G	0.6255	0.0159	0.0022	1.70E-13	387338	52.2
rs7927195	11	127478073	A	G	0.3847	0.0155	0.0021	1.90E-13	387338	54.5
rs543528	11	134099101	T	G	0.2764	-0.0125	0.0023	4.20E-08	387338	29.5
rs1074329	12	19149370	G	C	0.3743	-0.013	0.0021	6.50E-10	387338	38.3
rs1077045	12	19381384	T	C	0.1781	0.0149	0.0027	2.10E-08	387338	30.5
rs1104722	12	24195587	C	T	0.5609	0.0133	0.0021	1.80E-10	387338	40.1
rs1472874	12	24575272	T	C	0.8063	-0.0161	0.0028	1.30E-08	387338	33.1
rs285582	12	41948845	C	T	0.1788	0.0182	0.0028	2.80E-11	387338	42.2
rs5728106	12	54660427	G	A	0.5891	-0.0117	0.0021	1.30E-08	387338	31.0
rs7955865	12	56468706	A	T	0.3515	0.0134	0.0022	8.40E-10	387338	37.1
rs7972441	12	84043146	C	A	0.3575	0.0149	0.0022	1.10E-11	387338	45.9
rs2279574	12	89745477	C	A	0.4575	-0.0119	0.0021	2.60E-08	387338	32.1
rs7578337	13	27535824	T	C	0.8594	-0.0203	0.003	1.10E-10	387338	45.8
rs9554165	13	28393340	G	A	0.6306	0.0127	0.0022	5.30E-09	387338	33.3
rs9536961	13	55678332	A	G	0.5569	-0.0135	0.0022	1.90E-10	387338	37.7
rs6196082	13	57267105	G	A	0.9408	0.0255	0.0044	3.80E-09	387338	33.6
rs9538248	13	59492828	C	A	0.6783	0.0159	0.0022	3.00E-13	387338	52.2
rs341521	13	60399045	G	A	0.2978	0.0159	0.0023	9.00E-13	387338	47.8
rs2174752	13	69332015	G	T	0.5474	0.0128	0.0021	5.90E-10	387338	37.2
rs4144635	13	107653719	G	A	0.5030	-0.0115	0.0021	2.40E-08	387338	30.0
rs1289402	14	27611872	T	G	0.7145	0.0122	0.0023	3.80E-08	387338	28.1
rs1398814	14	30727612	A	T	0.9757	-0.0408	0.0068	1.20E-09	387338	36.0
rs1752212	14	33302882	T	G	0.6110	-0.0131	0.0023	1.80E-08	387338	32.4
rs1214746	14	41059928	G	A	0.8073	0.0202	0.0026	3.60E-14	387338	60.4
rs3007104	14	47367434	G	A	0.5767	0.0148	0.0021	1.50E-12	387338	49.7
rs1287835	14	58816212	A	C	0.4656	-0.0134	0.0021	5.30E-10	387338	40.7
rs1013765	14	85624262	G	T	0.6345	-0.0121	0.0022	1.60E-08	387338	30.2
rs1013469	14	93912668	C	A	0.4419	0.0143	0.0021	2.10E-11	387338	46.4
rs2892947	14	94844947	C	T	0.9799	-0.0416	0.0074	1.20E-08	387338	31.6
rs7671506	14	98597420	G	C	0.7919	0.0196	0.0026	1.90E-14	387338	56.8

rs1184798	14	103303514	T	C	0.7849	0.0208	0.0025	1.20E-16	387338	69.2
rs7166534	15	47678132	T	C	0.7873	0.0228	0.0025	1.50E-19	387338	83.2
rs783544	15	83240293	A	C	0.2505	-0.0148	0.0024	3.50E-10	387338	38.0
rs4702	15	91426560	G	A	0.4434	-0.0172	0.0021	1.10E-16	387338	67.1
rs1014403	15	97505901	G	A	0.5859	0.013	0.0021	8.00E-10	387338	38.3
rs763053	16	735921	T	C	0.7749	-0.0163	0.0025	4.90E-11	387338	42.5
rs9923553	16	5825579	A	G	0.7092	0.0139	0.0023	3.80E-10	387338	36.5
rs2870488	16	12350434	T	C	0.3243	-0.0125	0.0022	1.80E-08	387338	32.3
rs1107524	16	15116156	C	T	0.3838	-0.0126	0.0023	3.20E-08	387338	30.0
rs7188873	16	24727064	A	G	0.3768	0.0146	0.0021	5.80E-12	387338	48.3
rs1244873	16	49622284	C	T	0.8580	0.0179	0.003	2.10E-09	387338	35.6
rs7651377	16	72505534	T	C	0.8713	-0.024	0.0031	4.90E-15	387338	59.9
rs1244665	16	75607378	G	A	0.9451	-0.0266	0.0045	5.50E-09	387338	34.9
rs410520	17	4938924	C	T	0.4462	-0.0113	0.0021	4.60E-08	387338	29.0
rs3853548	17	21244607	A	G	0.4873	-0.0118	0.0021	4.10E-08	387338	31.6
rs2840636	17	47454507	C	T	0.6229	0.0165	0.0021	8.10E-15	387338	61.7
rs6504551	17	65903326	T	G	0.7363	0.0149	0.0023	1.10E-10	387338	42.0
rs7503604	17	79095629	C	A	0.4884	-0.0129	0.0021	6.10E-10	387338	37.7
rs4800204	18	22647270	C	T	0.4315	0.0125	0.0021	4.60E-09	387338	35.4
rs2849767	18	36959684	A	T	0.3252	0.0155	0.0022	4.60E-12	387338	49.6
rs5639397	18	39265197	G	T	0.8979	-0.0204	0.0034	1.70E-09	387338	36.0
rs1485443	18	40323567	C	T	0.9794	-0.0435	0.0073	2.50E-09	387338	35.5
rs3415504	18	44797697	C	T	0.5771	-0.0142	0.0021	1.80E-11	387338	45.7
rs2119839	18	52477074	G	A	0.3968	-0.0139	0.0021	2.80E-11	387338	43.8
rs9964201	18	50600552	C	A	0.9178	-0.0262	0.0038	4.20E-12	387338	47.5
rs1031831	18	53347258	C	A	0.6677	0.0155	0.0022	1.20E-12	387338	49.6
rs7236339	18	77579773	G	A	0.7714	0.0201	0.0025	3.90E-16	387338	64.6
rs1085398	19	4965064	G	A	0.6690	0.0123	0.0022	1.00E-08	387338	31.3
rs4804512	19	10707892	A	G	0.2351	-0.0137	0.0025	1.60E-08	387338	30.0
rs807478	19	36252494	A	G	0.5038	-0.0122	0.0021	1.90E-09	387338	33.8
rs1178311	20	14731057	C	T	0.9703	0.0356	0.0064	3.40E-08	387338	30.9
rs2145108	20	29457692	G	T	0.7408	-0.0133	0.0025	4.70E-08	387338	28.3
rs6058613	20	30864279	C	G	0.1615	0.0171	0.0028	1.80E-09	387338	37.3
rs211041	20	31932068	A	G	0.2949	0.0133	0.0023	2.70E-09	387338	33.4
rs1609598	20	51510926	C	T	0.6488	0.0122	0.0022	3.30E-08	387338	30.8
rs4809346	20	62434142	T	C	0.8424	-0.0179	0.003	1.20E-09	387338	35.6
rs7528133	21	40641262	T	C	0.9574	-0.0322	0.0058	3.10E-08	387338	30.8
rs7473421	23	53102956	A	T	0.8060	-0.0134	0.0022	1.50E-09	387338	37.1
rs6259979	23	101286946	G	A	0.8096	0.0122	0.0022	4.80E-08	387338	30.8
rs1468520	23	129118809	G	A	0.8594	-0.0165	0.0025	3.70E-11	387338	43.6
rs6637831	23	130423688	G	A	0.5280	-0.0178	0.0017	6.20E-25	387338	109.6
rs961522	2	58272502	T	C	0.3927	-0.0125	0.0021	8.60E-23	387338	35.4

rs7349386	2	213773970	T	C	0.9509	-0.0178	0.0048	1.50E-08	387338	13.8
rs7608187	2	50602587	G	A	0.5812	0.0112	0.0021	1.10E-10	387338	28.4
rs3448114	2	185607757	A	G	0.8520	-0.0138	0.0029	7.20E-09	387338	22.6
rs7024334	9	109072075	T	G	0.2203	-0.0157	0.0025	4.10E-14	387338	39.4
rs1103886	11	46355932	C	G	0.7133	0.0142	0.0023	8.90E-15	387338	38.1
rs7670207	11	85398919	T	A	0.9568	-0.0232	0.0051	1.30E-09	387338	20.7
rs590648	11	119753355	T	G	0.3149	-0.0091	0.0023	2.50E-08	387338	15.7
rs2039916	13	112238863	A	C	0.4729	0.0072	0.0021	3.90E-08	387338	11.8
rs1435757	15	47895902	C	A	0.4755	-0.0152	0.0021	3.10E-14	387338	52.4

Chr, chromosome; EA, effect allele; EAF, effect allele frequency; NEA, non-effect allele.

*N refers to the sample size of the initial GWAS from which the genetic variants were selected.

Table 3. Associations of single nucleotide polymorphisms for age at first birth.

**SNP**	**Chr**	**Position**	**EA**	**NEA**	**EAF**	**Beta**	**SE**	***p*-value**	**N**	**F-statistic**
rs1240743	1	22347396	A	G	0.8302	-0.0754	0.0138	4.37E-08	464846	29.9
rs2906457	1	44338575	A	C	0.2654	-0.0649	0.0103	2.92E-10	536976	39.7
rs2069278	1	66635371	T	C	0.6791	0.0576	0.0097	3.13E-09	536977	35.3
rs6677536	1	73837958	A	C	0.6213	0.0553	0.0101	3.85E-08	500306	30.0
rs1739169	1	78623626	T	C	0.1441	-0.083	0.0135	8.15E-10	527694	37.8
rs693691	1	88845494	T	C	0.137	0.077	0.0135	1.26E-08	541020	32.5
rs10559	1	153920564	G	A	0.3023	0.0804	0.0106	3.70E-14	475568	57.5
rs7516843	1	210322929	A	G	0.6832	-0.0611	0.0097	3.12E-10	541020	39.7
rs1188764	2	5891511	A	G	0.7631	-0.0707	0.0112	2.25E-10	505809	39.8
rs7277969	2	12797853	T	C	0.1222	-0.0849	0.0145	4.87E-09	530568	34.3
rs1606974	2	51873599	A	G	0.1325	0.0747	0.0137	4.61E-08	541022	29.7
rs1342073	2	53007398	A	T	0.3807	0.0595	0.0104	1.22E-08	302093	32.7
rs359247	2	60477052	A	T	0.3676	0.0682	0.0102	2.08E-11	475568	44.7
rs1189346	2	104158394	C	T	0.5223	0.0603	0.0095	2.26E-10	506256	40.3
rs8015328	2	156465747	A	C	0.0299	0.2314	0.0411	1.74E-08	514480	31.7
rs1122825	2	166250414	A	G	0.7545	-0.0631	0.0112	1.57E-08	500306	31.7
rs2116097	3	17912087	G	A	0.4569	-0.0603	0.0098	9.14E-10	475568	37.9
rs1125127	3	24905460	T	G	0.9114	-0.0965	0.0175	3.50E-08	530568	30.4
rs1171352	3	45422308	A	C	0.3085	0.0464	0.0098	1.14E-09	541022	22.4
rs6226208	3	47653822	T	C	0.2551	-0.0699	0.0107	7.09E-11	530568	42.7
rs3172494	3	48731487	T	G	0.1161	0.1341	0.0152	8.63E-19	503719	77.8
rs9838987	3	49916044	A	G	0.5039	0.1175	0.0098	5.55E-33	487702	143.8
rs1141428	3	51191466	T	C	0.0936	0.1077	0.0182	3.58E-09	491743	35.0
rs1191593	3	74883069	A	G	0.1828	0.073	0.0122	2.25E-09	510334	35.8
rs1452180	3	83504521	C	T	0.1877	-0.0673	0.0122	3.46E-08	506256	30.4
rs7623497	3	84666585	A	G	0.4264	-0.0583	0.01	5.03E-09	475568	34.0
rs6226174	3	85958954	C	G	0.6917	-0.0576	0.0104	2.85E-08	512484	30.7

rs9818010	3	132558241	T	G	0.2823	0.063	0.0102	5.63E-10	532196	38.1
rs6175081	4	77053834	T	C	0.8366	0.0796	0.0129	6.32E-10	524858	38.1
rs1731480	4	140946828	T	C	0.3567	0.0662	0.0095	2.67E-12	536237	48.6
rs3403355	4	159674014	G	C	0.6319	0.0603	0.0106	1.08E-08	457482	32.4
rs3392039	5	24900760	T	C	0.7232	0.0573	0.0104	3.93E-08	530568	30.4
rs1690206	5	45255478	T	A	0.1647	-0.0801	0.0129	5.92E-10	506256	38.6
rs1124222	5	133875033	A	G	0.7894	-0.0686	0.0122	1.67E-08	483177	31.6
rs1116775	5	140989410	T	C	0.7113	0.0574	0.01	1.04E-08	538858	32.9
rs1216394	5	155848785	T	C	0.6022	-0.0623	0.0103	1.76E-09	457484	36.6
rs6923535	6	19859654	A	G	0.4443	-0.052	0.0093	2.12E-08	530568	31.3
rs34137317	6	31604894	T	C	0.0277	0.2473	0.04	6.15E-10	468917	38.2
rs9276427	6	32711857	T	C	0.5217	0.0532	0.0096	3.29E-08	497401	30.7
rs1981258	6	63711033	T	C	0.6467	0.0586	0.0106	2.88E-08	457484	30.6
rs9372625	6	98344031	A	G	0.3672	-0.0719	0.0102	2.14E-12	475568	49.7
rs6925118	6	143165488	A	G	0.8064	-0.0676	0.0118	1.11E-08	530568	32.8
rs1220471	6	152235339	T	C	0.6361	-0.0891	0.0099	2.90E-19	506256	81.0
rs1464534	7	3505092	C	G	0.2965	-0.0606	0.0099	1.00E-09	541020	37.5
rs1773140	7	49854560	A	G	0.8464	0.0735	0.0129	1.21E-08	537447	32.5
rs7892866	7	71782460	A	G	0.9553	-0.142	0.0259	4.22E-08	529196	30.1
rs1132471	7	140182514	T	C	0.6961	-0.0641	0.0115	2.68E-08	461215	31.1
rs1124993	8	9556500	A	G	0.6107	0.0633	0.0099	1.56E-10	499880	40.9
rs3518028	8	91190780	C	T	0.2935	0.0642	0.0114	1.81E-08	457484	31.7
rs7865801	9	14745886	A	G	0.6329	0.0536	0.0097	3.35E-08	526995	30.5
rs1096255	9	16723742	T	C	0.1693	-0.0707	0.0128	3.12E-08	531520	30.5
rs1590949	9	23360417	C	G	0.5919	-0.0583	0.0095	8.24E-10	530568	37.7
rs1125359	9	140278206	A	C	0.1285	-0.0893	0.0163	4.45E-08	437423	30.0
rs4917916	10	102673292	A	G	0.2016	-0.0658	0.0119	3.48E-08	506256	30.6
rs1078683	10	106614571	G	A	0.6008	0.0604	0.0101	1.99E-09	475568	35.8
rs6585429	10	118893231	A	G	0.1877	-0.084	0.0124	1.08E-11	509779	45.9
rs4244533	11	28008972	A	G	0.7988	-0.0719	0.0119	1.33E-09	500807	36.5
rs1236159	11	30327006	A	G	0.1448	-0.0787	0.0142	2.78E-08	475568	30.7
rs933738	12	49943122	A	G	0.816	-0.0698	0.0122	1.10E-08	530568	32.7
rs1702877	12	56427808	T	C	0.3383	0.0602	0.0098	8.22E-10	530568	37.7
rs1077903	12	84109981	C	A	0.3594	0.0736	0.01	1.90E-13	506256	54.2
rs1281561	12	121407256	A	G	0.6653	-0.0564	0.0097	5.75E-09	536978	33.8
rs9540715	13	66913378	A	C	0.4645	0.0588	0.01	4.74E-09	475546	34.6
rs9561311	13	93978321	G	A	0.1468	0.0821	0.0147	2.29E-08	457484	31.2
rs6574018	14	72213258	T	G	0.3623	0.0556	0.01	2.42E-08	499879	30.9
rs2224234	14	103382038	A	C	0.7967	0.0851	0.0124	6.64E-12	466188	47.1
rs8030494	15	74092777	A	C	0.606	0.0545	0.0096	1.30E-08	511260	32.2
rs1121780	17	29261788	T	C	0.1893	-0.0677	0.0122	3.25E-08	506256	30.8
rs5743204	17	29241038	T	C	0.8982	-0.0905	0.0165	4.57E-08	499880	30.1

rs1044536	17	43932797	C	G	0.1843	-0.0886	0.0136	6.69E-11	453938	42.4
rs7359501	17	56641200	T	C	0.4091	0.0565	0.0094	1.82E-09	530568	36.1
rs6022268	18	36510587	T	C	0.2037	-0.0646	0.0117	3.51E-08	530568	30.5
rs590076	18	53260732	A	G	0.3505	-0.0553	0.0095	5.78E-09	536239	33.9
rs8110682	19	13285293	T	C	0.6348	-0.0695	0.011	2.59E-10	457959	39.9
rs293566	20	31097877	T	C	0.6472	0.0694	0.0099	2.18E-12	505549	49.1
rs2253763	21	46642764	T	C	0.4001	0.0534	0.0098	4.58E-08	494963	29.7
rs5763436	22	30121577	A	T	0.5902	0.0542	0.0097	1.94E-08	492248	31.2
rs5932889	23	130436456	C	G	0.6473	-0.0896	0.0119	4.88E-14	431450	56.7
rs183103	23	131768905	T	C	0.7001	0.0887	0.0122	4.27E-13	431450	52.9
rs1283717	23	51117172	A	C	0.8139	0.083	0.0146	1.38E-08	426927	32.3
rs1442451	23	136982365	A	G	0.0826	0.1629	0.0295	3.46E-08	422166	30.5

Chr, chromosome; EA, effect allele; EAF, effect allele frequency; NEA, non-effect allele.

Table 4. Proxies used for SNPs that was not available for an outcome.

**SNP**	**Proxies**	**R^2**	**SNP**	**Proxies**	**R^2**
**Age at first sex**			**Age at first birth**		
rs111991969	None		rs34137317	None	
rs199769930	None		rs5932889	None	
rs577722844	None		rs183103	None	
rs10137654	None		rs12837176	None	
rs7473421	None		rs144245162	None	
rs62599791	None		rs112535944	None	
rs146852038	None		rs80153284	None	
rs6637831	None		rs9276427	None	
rs79764489	None		rs11242222	rs62381443	0.697
rs73581580	None		rs35180283	rs11776764	0.995
rs28929474	None				
rs148544378	None				
rs1146566	rs1222923	0.984			
rs12878359	rs12879717	0.779			
rs1014403	rs1014402	0.996			
rs4809346	rs4809345	0.958			
rs245753	rs245754	1			
rs372420182	rs147678290	1			
rs7943198	rs4294596	0.996			
rs2145108	rs6118976	0.927			
rs2039916	rs6492373	1			
rs3853548	rs16942379	0.861			

Table 5. Mendelian randomization association of genetically predicted age at first sex with cardiovascular diseases.

Cardiovascular disease	Weighted median method			MR-Egger regression method				
	OR	95% CI	*p*-value	OR	95% CI	*p*-value	Intercept	*p*-value
Heart failure	0.746	0.665, 0.837	6.17E-07	0.875	0.600, 1.275	0.487	-0.004	0.234
Coronary artery disease	0.697	0.611, 0.796	9.94E-08	0.807	0.526, 1.236	0.325	-0.002	0.629
Myocardial infarction	0.721	0.624, 0.834	9.72E-06	0.710	0.456, 1.105	0.130	4.57E-04	0.897
Stroke	0.778	0.691, 0.877	3.60E-05	0.990	0.686, 1.427	0.955	-0.005	0.122
Atrial fibrillation	0.903	0.816, 1.000	0.049	1.003	0.726, 1.386	0.985	-0.002	0.377

Table 6. Heterogeneity test by using MR Egger method for each outcome.

Exposure	Outcomes	Heterogeneity			
		Q	O_df	*p*-value	I^2 (%)
Age at first sex	Heart failure	378.1	249	2.37E-07	34.1
	Coronary artery disease	352.3	248	1.45E-05	29.6
	Myocardial infarction	307.5	248	0.006	19.3
	Stroke	336.5	248	1.54E-04	26.3
	Atrial fibrillation	343.2	249	6.90E-05	27.4

Table 7. Mendelian randomization association of genetically predicted age at first sex with ischemic stroke and ischemic stroke subtypes.

Exposure	Outcome	Method	OR	95% CI	*p*-value
Age at first sex	Any ischemic stroke	Inverse-variance weighted	0.747	0.682, 0.819	3.75E-10
		Weighted median	0.784	0.695, 0.884	6.91E-05
		MR Egger	0.941	0.643, 1.377	0.754
	large-artery stroke	Inverse-variance weighted	0.669	0.545, 0.821	1.21E-04
		Weighted median	0.714	0.536, 0.951	0.021
		MR Egger	0.461	0.195, 1.093	0.080
	Small-vessel stroke	Inverse-variance weighted	0.755	0.639, 0.893	9.87E-04
		Weighted median	0.821	0.641, 1.053	0.120
		MR Egger	0.999	0.484, 2.061	0.998
	Cardioembolic stroke	Inverse-variance weighted	0.830	0.699, 0.986	0.034
		Weighted median	0.873	0.677, 1.126	0.297
		MR Egger	1.660	0.803, 3.434	0.173

Table 8. The genetic liability to age at first sex with cardiovascular risk factors.

**Exposure**	**Outcomes**	**SNP**	**IVW OR (95% CI)**	***p*-value**
Age at first sex	Smoking	243	0.487 (0.455, 0.520)	2.54E-101
	Alcohol intake	243	0.892 (0.867, 0.918)	3.97E-15
	Body mass index	196	0.755 (0.705, 0.809)	1.03E-15
	Depression	237	0.702 (0.656, 0.751)	1.38E-24
	Education attainment	221	1.548 (1.491, 1.608)	5.54E-115

Table 9. Mediation effect of education attainment on age at first sex-CVDs association.

**Outcomes**	**Total Effect**	**Mediation Effect**		**Mediated Proportion**
	**Effect Size (95%CI)**	**Effect Size (95%CI)**	***p*-value**	**(%) (95% CI)**
**Age at first sex**				
Heart failure	-0.356 (-0.447, -0.265)	-0.080 (-0.142 -0.019)	0.010	22.6 (5.4, 39.8)
Coronary artery disease	-0.317 (-0.420, -0.214)	-0.134 (-0.211 -0.058)	5.64E-04	42.3 (18.3, 66.4)
Myocardial infarction	-0.314 (-0.421, -0.207)	-0.139 (-0.220 -0.058)	8.19E-04	44.3 (18.4, 70.3)
Stroke	-0.292 (-0.379, -0.204)	-0.102 (-0.163 -0.041)	9.89E-04	34.9 (14.2, 55.7)
Atrial fibrillation	-0.139 (-0.216, -0.061)	0.002 (-0.065 0.069)	0.959	-1.3 (-49.5, 46.9)

Table 10. Associations of single nucleotide polymorphisms for age at first sex, in female only.

**SNP**	**Chr**	**Position**	**EA**	**NEA**	**EAF**	**Beta**	**SE**	***p*-value**	**N***	**F-statistic**
rs141655075	1	87794596	C	T	0.9611	0.0424	0.0072	5.10E-09	214,547	34.7
rs11240331	1	204968339	C	T	0.7383	-0.0185	0.0032	5.10E-09	214,547	33.8
rs67446571	2	22554648	A	G	0.6304	0.0174	0.0029	1.20E-09	214,547	36.6
rs56254887	2	44842184	T	A	0.3797	0.0177	0.0028	9.30E-10	214,547	38.5
rs9789483	2	58492345	A	G	0.6351	0.0274	0.0029	1.80E-21	214,547	90.6
rs6719762	2	60166832	T	C	0.5258	0.0245	0.0028	6.90E-19	214,547	77.7
rs359243	2	60475509	T	C	0.3918	0.0205	0.0028	6.30E-13	214,547	51.7
rs12328348	2	60785937	A	G	0.5583	-0.0162	0.0028	1.40E-08	214,547	32.5
rs62180269	2	63320524	T	C	0.7815	-0.0195	0.0034	4.10E-09	214,547	33.8
rs11678980	2	162101261	G	A	0.5374	0.0156	0.0029	3.30E-08	214,547	29.8
rs7575189	2	174015168	G	A	0.4108	-0.0176	0.0028	2.30E-10	214,547	39.1
rs67723420	3	35775115	T	A	0.6251	-0.0166	0.0029	4.30E-09	214,547	33.5
rs111599092	3	49502779	A	G	0.1147	0.0353	0.0045	1.10E-14	214,547	60.5
rs2188151	3	50201924	G	T	0.5753	0.0190	0.0028	1.10E-11	214,547	45.9
rs186723454	3	54156598	A	G	0.8491	0.0222	0.0039	1.80E-08	214,547	32.5
rs12714592	3	84387950	A	C	0.7265	0.0242	0.0031	7.50E-15	214,547	60.9
rs112523595	3	85650341	T	C	0.3707	-0.0200	0.0029	5.00E-12	214,547	47.8
rs12714702	3	88249922	A	G	0.1596	0.0247	0.0038	7.50E-11	214,547	43.1
rs1377276	3	107948019	C	G	0.4161	0.0181	0.0028	1.50E-10	214,547	40.3
rs57945129	3	117641724	C	T	0.8514	-0.0236	0.0039	1.40E-09	214,547	36.6

rs16859457	3	147161075	A	G	0.6151	-0.0199	0.0031	3.10E-10	214,547	40.5
rs11729080	4	112503872	G	A	0.8283	-0.0224	0.0037	1.10E-09	214,547	37.4
rs809955	4	140874760	G	A	0.6332	-0.0172	0.0029	1.20E-09	214,547	35.8
rs2910032	5	152540354	C	T	0.4813	-0.0174	0.0028	2.40E-10	214,547	39.2
rs12203592	6	396321	C	T	0.7806	-0.0206	0.0034	4.60E-09	214,547	35.9
rs12204714	6	152235339	C	T	0.3679	-0.0288	0.0029	6.30E-24	214,547	100.1
rs6951209	7	1273821	T	A	0.2253	0.0214	0.0034	3.50E-10	214,547	39.5
rs4552798	7	1928001	C	T	0.4618	-0.0182	0.0029	1.30E-10	214,547	40.6
rs7785195	7	3424686	G	A	0.3415	-0.0167	0.0029	1.10E-08	214,547	32.9
rs198310	7	24191861	A	T	0.7854	-0.0200	0.0034	3.50E-09	214,547	34.8
rs6962772	7	99081730	A	G	0.8460	-0.0253	0.0039	9.70E-11	214,547	43.0
rs7783012	7	114116881	G	A	0.4070	0.0163	0.0028	8.10E-09	214,547	33.0
rs11770163	7	116417848	G	C	0.6685	0.0167	0.0029	2.20E-08	214,547	32.1
rs113367286	7	140144414	C	T	0.7208	-0.0172	0.0031	1.20E-08	214,547	31.0
rs6997641	8	86676101	C	T	0.1738	-0.0224	0.0037	4.90E-10	214,547	37.4
rs3896224	10	106467853	A	G	0.5879	-0.0210	0.0028	7.00E-14	214,547	54.6
rs10749233	10	118777998	G	C	0.2361	-0.0195	0.0033	7.30E-09	214,547	34.4
rs7079070	10	134182921	G	A	0.5461	0.0164	0.0028	4.50E-09	214,547	34.9
rs10835387	11	28671629	G	C	0.6557	-0.0177	0.0029	1.60E-09	214,547	36.9
rs34804222	11	43771084	A	G	0.5935	-0.0164	0.0028	2.90E-09	214,547	33.8
rs1472874	12	24575272	T	C	0.8059	-0.0210	0.0037	2.50E-08	214,547	31.9
rs61960829	13	57267105	G	A	0.9412	0.0324	0.0059	4.40E-08	214,547	29.9
rs12147463	14	41059928	G	A	0.8072	0.0269	0.0036	5.20E-14	214,547	57.4
rs3007104	14	47367434	G	A	0.5768	0.0152	0.0028	4.50E-08	214,547	29.2
rs76715069	14	98597420	G	C	0.7918	0.0226	0.0034	2.10E-11	214,547	43.4
rs11847989	14	103303514	T	C	0.7848	0.0201	0.0034	1.70E-09	214,547	35.1
rs7166534	15	47678132	T	C	0.7868	0.0202	0.0034	3.10E-09	214,547	35.3
rs76513770	16	72505534	T	C	0.8714	-0.0234	0.0041	8.50E-09	214,547	31.8
rs28406364	17	47454507	C	T	0.6219	0.0200	0.0029	2.40E-12	214,547	48.5
rs2849767	18	36959684	A	T	0.3254	0.0189	0.0030	3.20E-10	214,547	39.7
rs148544378	18	40323567	C	T	0.9793	-0.0551	0.0097	2.10E-08	214,547	32.1
rs1031831	18	53351539	C	A	0.6683	0.0178	0.0029	1.00E-09	214,547	36.6
rs7236339	18	77579773	G	A	0.7722	0.0196	0.0033	2.40E-09	214,547	34.7
rs6637831	23	130423688	G	A	0.5279	-0.0213	0.0028	2.20E-14	214,547	58.7
rs961522	2	58272502	T	C	0.3925	-0.0278	0.0028	8.60E-23	214,547	96.2
rs7349386	2	213773970	T	C	0.9509	-0.0369	0.0065	1.50E-08	214,547	32.6

Chr, chromosome; EA, effect allele; EAF, effect allele frequency; NEA, non-effect allele.

*N refers to the sample size of the initial GWAS from which the genetic variants were selected.

Table 11. Associations of single nucleotide polymorphisms for age at first sex, in male only.

SNP	Chr	Position	EA	NEA	EAF	Beta	SE	*p*-value	N*	F-statistic
rs35851551	7	31330785	A	G	0.8986	0.0301	0.0052	7.70E-09	182,791	33.5
rs12701263	7	32962089	C	T	0.6006	-0.0181	0.0032	4.60E-09	182,791	32.0
rs794375	7	75147801	T	C	0.5741	-0.0203	0.0032	1.20E-10	182,791	40.2
rs13307225	7	105084671	G	A	0.1068	-0.0293	0.005	8.10E-09	182,791	34.3
rs7783012	7	114116881	G	A	0.4075	0.0235	0.0032	1.40E-13	182,791	53.9
rs11767283	7	121947456	A	G	0.779	0.0222	0.0038	3.20E-09	182,791	34.1
rs11772444	7	133453874	G	A	0.8101	0.0246	0.004	5.60E-10	182,791	37.8
rs6966898	7	135221170	C	T	0.6674	0.0183	0.0033	3.70E-08	182,791	30.8
rs113367286	7	140144414	C	T	0.7206	-0.0194	0.0035	2.90E-08	182,791	30.7
rs1991651	8	10706411	C	G	0.3814	-0.0208	0.0032	5.10E-11	182,791	42.2
rs35918434	8	91894514	T	C	0.7432	0.02	0.0035	1.90E-08	182,791	32.7
rs13280592	8	116686752	C	G	0.2732	0.0195	0.0035	1.40E-08	182,791	31.0
rs2090409	9	108967088	C	A	0.6516	0.0231	0.0033	2.70E-12	182,791	49.0
rs61856978	10	97941022	T	C	0.6604	0.0202	0.0033	2.50E-10	182,791	37.5
rs3896224	10	106467853	A	G	0.5876	-0.0189	0.0032	2.20E-09	182,791	34.9
rs61882758	11	46611805	A	G	0.7387	0.0267	0.0039	8.10E-12	182,791	46.9
rs11040367	11	49450176	G	A	0.1487	-0.0262	0.0047	2.50E-08	182,791	31.1
rs7943198	11	112882179	T	G	0.6249	0.0191	0.0032	3.90E-09	182,791	35.6
rs7955865	12	56468706	A	T	0.3522	0.0179	0.0032	3.20E-08	182,791	31.3
rs341521	13	60399045	G	A	0.297	0.0194	0.0034	1.10E-08	182,791	32.6
rs11847989	14	103303514	T	C	0.7849	0.0212	0.0038	1.00E-08	182,791	31.1
rs7166534	15	47678132	T	C	0.788	0.0263	0.0038	3.30E-12	182,791	47.9
rs4702	15	91426560	G	A	0.4431	-0.0213	0.0031	7.10E-12	182,791	47.2
rs7188873	16	24727064	A	G	0.3753	0.019	0.0032	4.10E-09	182,791	35.3
rs76513770	16	72505534	T	C	0.8711	-0.0264	0.0046	1.90E-08	182,791	32.9
rs2119839	18	52477074	G	A	0.3965	-0.0172	0.0032	2.80E-08	182,791	28.9
rs7236339	18	77579773	G	A	0.7704	0.0224	0.0037	2.40E-09	182,791	36.7
rs7473421	23	53102956	A	T	0.8054	-0.0172	0.0028	1.10E-09	182,791	37.7
rs146852038	23	129118809	G	A	0.8589	-0.0177	0.0032	4.10E-08	182,791	30.6
rs6637831	23	130423688	G	A	0.528	-0.0157	0.0022	8.10E-13	182,791	50.9
rs7608187	2	50602587	G	A	0.5815	0.0201	0.0031	1.10E-10	182,791	42.0
rs34481141	2	185607757	A	G	0.8511	-0.0253	0.0043	7.20E-09	182,791	34.6
rs7024334	9	109072075	T	G	0.2212	-0.0286	0.0037	4.10E-14	182,791	59.7
rs11038866	11	46355932	C	G	0.7142	0.0267	0.0034	8.90E-15	182,791	61.7
rs76702070	11	85398919	T	A	0.9567	-0.0469	0.0077	1.30E-09	182,791	37.1
rs590648	11	119753355	T	G	0.3147	-0.0191	0.0034	2.50E-08	182,791	31.6
rs2039916	13	112238863	A	C	0.4724	0.0173	0.0031	3.90E-08	182,791	31.1
rs1435757	15	47895902	C	A	0.4741	-0.0238	0.0031	3.10E-14	182,791	58.9

Chr, chromosome; EA, effect allele; EAF, effect allele frequency; NEA, non-effect allele.

*N refers to the sample size of the initial GWAS from which the genetic variants were selected.

Table 12. Mendelian randomization association of genetically predicted age at first sex with cardiovascular diseases, in female only.

Exposure	Outcome	Method	OR	95% CI	*p*-value
Age at first sex (Female only)	Heart failure	Inverse-variance weighted	0.717	0.606, 0.848	9.92E-05
		Weighted median	0.812	0.670, 0.984	0.033
		MR Egger	1.748	0.835, 3.662	0.145
	Myocardial infarction	Inverse-variance weighted	0.726	0.617, 0.855	1.21E-04
		Weighted median	0.820	0.655, 1.026	0.083
		MR Egger	0.812	0.377, 1.751	0.598
	Coronary artery disease	Inverse-variance weighted	0.762	0.649, 0.896	0.001
		Weighted median	0.896	0.727, 1.103	0.301
		MR Egger	0.926	0.432, 1.982	0.843
	Stroke	Inverse-variance weighted	0.789	0.695, 0.896	2.57E-04
		Weighted median	0.807	0.676, 0.963	0.017
		MR Egger	1.331	0.729, 2.431	0.356
	Atrial fibrillation	Inverse-variance weighted	0.853	0.753, 0.966	0.012
		Weighted median	0.834	0.711, 0.979	0.027
		MR Egger	1.704	0.970, 2.991	0.069

Table 13. Mendelian randomization association of genetically predicted age at first sex with cardiovascular diseases, in male only.

Exposure	Outcome	Method	OR	95% CI	*p*-value
Age at first sex (Male only)	Heart failure	Inverse-variance weighted	0.706	0.582, 0.855	3.80E-04
		Weighted median	0.683	0.549, 0.851	6.56E-04
		MR Egger	0.529	0.176, 1.587	0.264
	Myocardial infarction	Inverse-variance weighted	0.804	0.646, 1.001	0.051
		Weighted median	0.824	0.631, 1.076	0.156
		MR Egger	0.843	0.238, 2.988	0.792
	Coronary artery disease	Inverse-variance weighted	0.857	0.706, 1.041	0.119
		Weighted median	0.856	0.665, 1.102	0.229
		MR Egger	0.888	0.290, 2.725	0.837
	Stroke	Inverse-variance weighted	0.669	0.546, 0.819	1.02E-04
		Weighted median	0.660	0.525, 0.831	3.97E-04
		MR Egger	0.689	0.211, 2.250	0.542
	Atrial fibrillation	Inverse-variance weighted	0.956	0.843, 1.084	0.482
		Weighted median	0.939	0.784, 1.125	0.492
		MR Egger	1.371	0.665, 2.827	0.398

Table 14. Mendelian randomization association of genetically predicted age at first birth with cardiovascular diseases.

**Cardiovascular disease**	**Weighted median method**			**MR-Egger regression method**				
	**OR**	**95% CI**	***p*-value**	**OR**	**95% CI**	***p*-value**	**Intercept**	***p*-value**
Myocardial infarction	0.923	0.868, 0.982	0.011	0.811	0.642, 1.025	0.084	0.007	0.403
Coronary artery disease	0.933	0.880, 0.990	0.021	0.749	0.602, 0.931	0.011	0.013	0.092
Heart failure	0.973	0.924, 1.024	0.296	0.923	0.773, 1.102	0.379	1.68E-04	0.978
Atrial fibrillation	0.967	0.926, 1.010	0.131	0.982	0.835, 1.154	0.827	-0.003	0.623
Stroke	0.967	0.919, 1.017	0.190	1.007	0.832, 1.220	0.941	-0.003	0.674

Table 15. Heterogeneity test by using MR Egger method for each outcome.

**Exposure**	**Outcomes**	**Heterogeneity**			
		**Q**	**O_df**	***p*-value**	**I^2 (%)**
Age at first birth	Myocardial infarction	102.3	72	0.011	29.6
	Coronary artery disease	107.9	72	0.004	33.3
	Heart failure	106.7	74	0.008	30.6
	Atrial fibrillation	124.8	75	2.70E-04	39.9
	Stroke	101.7	73	0.015	28.2

Table 16. The genetic liability to age at first birth with cardiovascular risk factors.

**Exposure**	**Outcomes**	**SNP**	**IVW OR (95% CI)**	***p*-value**
Age at first birth	Smoking	74	0.900 (0.869, 0.933)	8.42E-09
	Alcohol intake	75	1.000 (0.987, 1.012)	0.940
	Body mass index	55	0.945 (0.921, 0.970)	1.91E-05
	Depression	72	0.925 (0.892, 0.958)	1.67E-05
	Education attainment	66	1.086 (1.054, 1.118)	5.14E-08

Table 17. Mediation effect of education attainment on age at first birth-CVDs association.

**Outcomes**	**Total Effect**	**Mediation Effect**		**Mediated Proportion**
	**Effect Size (95%CI)**	**Effect Size (95%CI)**	***p*-value**	**(%) (95% CI)**
**Age at first birth**				
Myocardial infarction	-0.111 (-0.160, -0.062)	-0.026 (-0.046, -0.007)	0.008	23.9 (6.4, 41.4)
Coronary artery disease	-0.104 (-0.150, -0.058)	-0.027 (-0.045, -0.008)	0.005	25.5 (7.8, 43.3)
Heart failure	-0.078 (-0.116, -0.040)	-0.020 (-0.034, -0.005)	0.009	25.4 (6.4, 44.3)
Atrial fibrillation	-0.058 (-0.093, -0.022)	-0.006 (-0.020, 0.009)	0.444	9.7 (-15.1, 34.5)

Table 18. Associations of single nucleotide polymorphisms for age at first birth, in female only.

SNP	Chr	Position	EA	NEA	EAF	Beta	SE	*p*-value	N	F-statistic
rs12407439	1	22347396	A	G	0.8311	-0.0876	0.0151	6.86E-09	343903	33.7
rs2906457	1	44338575	A	C	0.2638	-0.0677	0.0112	1.43E-09	412834	36.5
rs2069278	1	66635371	T	C	0.6798	0.0592	0.0105	1.87E-08	412834	31.8
rs10559	1	153920564	G	A	0.3039	0.0892	0.0116	1.14E-14	357353	59.1
rs7516843	1	210322929	A	G	0.6833	-0.0623	0.0105	3.37E-09	416877	35.2
rs11887646	2	5891511	A	G	0.7642	-0.077	0.0121	2.14E-10	383785	40.5
rs72779695	2	12797853	T	C	0.1198	-0.087	0.0158	3.76E-08	406425	30.3
rs1606974	2	51873599	A	G	0.1297	0.0828	0.0149	2.51E-08	416879	30.9
rs359247	2	60477052	A	T	0.3676	0.0856	0.0111	1.14E-14	357353	59.5
rs11893460	2	104158394	C	T	0.5235	0.0711	0.0103	5.33E-12	388041	47.6
rs80153284	2	156465747	A	C	0.0231	0.2459	0.0439	2.17E-08	396518	31.4
rs112282597	2	166250414	A	G	0.7538	-0.0703	0.0122	8.37E-09	377120	33.2
rs2116097	3	17912087	G	A	0.458	-0.0631	0.0107	4.14E-09	357353	34.8
rs62262084	3	47653822	T	C	0.2536	-0.0715	0.0117	8.37E-10	406425	37.3
rs3172494	3	48731487	T	G	0.114	0.1412	0.0164	8.01E-18	380898	74.1
rs9838987	3	49916044	A	G	0.498	0.1268	0.0107	2.34E-32	364516	140.4
rs114142815	3	51191466	T	C	0.0908	0.108	0.0196	3.74E-08	370636	30.4
rs11915934	3	74883069	A	G	0.1811	0.0826	0.0133	4.75E-10	386191	38.6
rs1452180	3	83504521	C	T	0.1875	-0.0801	0.0132	1.42E-09	388041	36.8
rs17314804	4	140946828	T	C	0.3565	0.0736	0.0103	7.79E-13	412095	51.1
rs16902068	5	45255478	T	A	0.1662	-0.091	0.014	7.57E-11	388041	42.2
rs34137317	6	32092625	T	C	0.0251	0.2429	0.042	7.06E-09	357311	33.4
rs9276427	6	32711857	T	C	0.5193	0.0606	0.0105	7.44E-09	377409	33.3
rs9372625	6	98344031	A	G	0.3667	-0.0701	0.0112	3.26E-10	357353	39.2
rs6925118	6	143165488	A	G	0.8077	-0.0714	0.0129	2.75E-08	406425	30.6
rs12204714	6	152235339	T	C	0.6382	-0.09	0.0108	7.00E-17	388041	69.4
rs1464534	7	3505092	C	G	0.2944	-0.0615	0.0108	1.15E-08	416877	32.4
rs11249939	8	9556500	A	G	0.6134	0.063	0.0108	5.28E-09	375737	34.0
rs35180283	8	91190780	C	T	0.2916	0.0682	0.0125	4.48E-08	339269	29.8
rs7865801	9	14745886	A	G	0.6331	0.0651	0.0105	6.38E-10	404971	38.4
rs10962552	9	16723742	T	C	0.1685	-0.0753	0.0138	4.77E-08	407377	29.8
rs1590949	9	23360417	C	G	0.5916	-0.069	0.0103	2.15E-11	406425	44.9
rs10786831	10	106614571	G	A	0.6018	-0.073	0.011	2.85E-11	357353	44.0
rs6585429	10	118893231	A	G	0.1854	-0.0956	0.0134	9.03E-13	386245	50.9
rs12361594	11	30327006	A	G	0.146	-0.0902	0.0153	3.83E-09	357353	34.8
rs1702877	12	56427808	T	C	0.3369	0.0628	0.0107	3.90E-09	406425	34.4
rs10779030	12	84107863	C	A	0.3621	0.0837	0.0109	1.26E-14	388041	59.0
rs9561311	13	93978321	G	A	0.1431	0.0925	0.0162	1.15E-08	339269	32.6
rs6574018	14	72213258	T	G	0.3607	0.0601	0.0109	3.24E-08	375737	30.4
rs2224234	14	103382038	A	C	0.7964	0.0876	0.0134	7.22E-11	352409	42.7

rs8030494	15	74092777	A	C	0.6065	0.0575	0.0104	3.66E-08	387117	30.6
rs10445366	17	43932797	C	G	0.1821	-0.0888	0.0149	2.61E-09	334705	35.5
rs7359501	17	56641200	T	C	0.4067	0.0625	0.0102	1.01E-09	406425	37.5
rs60222682	18	36510587	T	C	0.2026	-0.0724	0.0127	1.21E-08	406425	32.5
rs590076	18	53260732	A	G	0.3494	-0.0665	0.0103	1.06E-10	412096	41.7
rs8110682	19	13285293	T	C	0.6326	-0.0873	0.0119	2.59E-13	341109	53.8
rs293566	20	31097877	T	C	0.6498	0.071	0.0108	4.42E-11	381406	43.2
rs2253763	21	46642764	T	C	0.3998	0.0583	0.0107	4.75E-08	373601	29.7
rs5932889	23	130436456	C	G	0.6522	-0.087	0.013	2.21E-11	318063	44.8
rs183103	23	131768905	T	C	0.6975	0.0954	0.0132	5.23E-13	318063	52.2
rs11713522	3	45422308	A	C	0.3079	0.0646	0.0106	1.14E-09	416879	37.1

Chr, chromosome; EA, effect allele; EAF, effect allele frequency; NEA, non-effect allele.

Table 19. Mendelian randomization association of genetically predicted age at first birth with cardiovascular diseases, in female only.

**Exposure**	**Outcome**	**Method**	**OR**	**95% CI**	***p*-value**
Age at first birth (Female only)	Coronary artery disease	Inverse-variance weighted	0.928	(0.879, 0.980)	0.007
		Weighted median	0.970	(0.908, 1.036)	0.362
		MR Egger	0.727	(0.566, 0.934)	0.016
	Myocardial infarction	Inverse-variance weighted	0.928	(0.876, 0.984)	0.012
		Weighted median	0.940	(0.877, 1.008)	0.082
		MR Egger	0.759	(0.578, 0.996)	0.053
	Heart failure	Inverse-variance weighted	0.932	(0.892, 0.973)	0.001
		Weighted median	0.967	(0.917, 1.020)	0.223
		MR Egger	0.940	(0.764, 1.156)	0.561
	Atrial fibrillation	Inverse-variance weighted	0.943	(0.906, 0.981)	0.004
		Weighted median	0.971	(0.926, 1.017)	0.056
		MR Egger	1.070	(0.899, 1.275)	0.216
	Stroke	Inverse-variance weighted	0.981	(0.941, 1.023)	0.373
		Weighted median	1.022	(0.969, 1.077)	0.425
		MR Egger	1.121	(0.908, 1.384)	0.293
